# Where We Used to Live: Validating Retrospective Measures of Childhood Neighborhood Context for Life Course Epidemiologic Studies

**DOI:** 10.1371/journal.pone.0124635

**Published:** 2015-04-21

**Authors:** Theresa L. Osypuk, Rebecca Kehm, Dawn P. Misra

**Affiliations:** 1 University of Minnesota School of Public Health, Division of Epidemiology & Community Health, Minneapolis, Minnesota, United States of America; 2 Wayne State University School of Medicine, Department of Family Medicine and Public Health Sciences, Detroit, Michigan, United States of America; Institute of Psychiatry, UNITED KINGDOM

## Abstract

Early life exposures influence numerous social determinants of health, as distal causes or confounders of later health outcomes. Although a growing literature is documenting how early life socioeconomic position affects later life health, few epidemiologic studies have tested measures for operationalizing early life neighborhood context, or examined their effects on later life health. In the Life-course Influences on Fetal Environments (LIFE) Study, a retrospective cohort study among Black women in Southfield, Michigan (71% response rate), we tested the validity and reliability of retrospectively-reported survey-based subjective measures of early life neighborhood context(N=693). We compared 3 subjective childhood neighborhood measures (disorder, informal social control, victimization), with 3 objective childhood neighborhood measures derived from 4 decades of historical census tract data 1970-2000, linked through geocoded residential histories (tract % poverty, tract % black, tract deprivation score derived from principal components analysis), as well as with 2 subjective neighborhood measures in adulthood. Our results documented that internal consistency reliability was high for the subjective childhood neighborhood scales (Cronbach’s α =0.89, 0.93). Comparison of subjective with objective childhood neighborhood measures found moderate associations in hypothesized directions. Associations with objective variables were strongest for neighborhood disorder (rhos=.40), as opposed to with social control or victimization. Associations between subjective neighborhood context in childhood versus adulthood were moderate and stronger for residentially-stable populations. We lastly formally tested for, but found little evidence of, recall bias of the retrospective subjective reports of childhood context. These results provide evidence that retrospective reports of subjective neighborhood context may be a cost-effective, valid, and reliable method to operationalize early life context for health studies.

## Introduction

A growing social epidemiology literature is documenting that adverse neighborhood and place-related context is associated with a range of adverse health and developmental consequences. [1–4] A life course epidemiology literature has simultaneously evolved by documenting the effects of early life exposures on later life health. [5–7] However these literatures have evolved separately. Indeed, although context refers to both place and time, [7] the health literature with respect to context typically operationalizes either place or time. Incorporating both place and time for understanding causes of disease is one of the key challenges for epidemiologic research.

Although neighborhood-health studies have been improving over the last decade, few studies have examined effects of early life course neighborhood context on health.[8] Early life neighborhood environment may play an important role for patterning early life exposures that may influence health outcomes later in life.[8] Yet most neighborhood-health studies refer to the current neighborhood context of adults. Though longitudinal neighborhood-health studies are becoming more prevalent, [4] the neighborhood exposure window typically references a discrete period of time in adulthood [9,10] instead of childhood for associations with later-life disease. The contemporaneous neighborhood-health literature has shifted away from using solely census-based demographic and compositional variables, towards using subjective measures of the neighborhood social environment to operationalize neighborhood environment. [4] It follows that life course neighborhood studies would benefit from this shift as well.

The purpose of this manuscript is to test the validity and reliability of measuring a respondent’s childhood neighborhood context retrospectively with subjective reports of neighborhood social environment elicited in adulthood. We compare these subjective reports to objective neighborhood measures of childhood context, derived from residential histories and geocoded to historical census data, including to formally test for measurement recall bias. We also compare to adulthood neighborhood context. Our larger goal is to identify more efficient methods to operationalize the early life neighborhood context than those from prospective cohort studies begun in childhood, or from administrative record linkage, and thereby provide tools to advance the social and life course epidemiology literatures to incorporate context of both place and time. As discussed below, our results provide evidence that retrospective reports of subjective neighborhood context may be a cost-effective, valid, and reliable method to operationalize early life context.

## Methods

The Life-course Influences on Fetal Environments (LIFE) study is a retrospective cohort study of self-reported Black/African American women aged 18–45 who had just given birth to a singleton baby in a Detroit, MI suburban hospital (Providence Hospital, Southfield MI). Women were recruited from the hospital’s labor and delivery and postpartum unit logs. All eligible women were approached for study enrollment during postpartum hospitalization, and written informed consent was obtained upon enrollment. Trained interviewers conducted interviews in women’s hospital rooms after delivery during the immediate postpartum hospitalization. A $50 gift card to a local store was provided as an interview incentive. Enrollment for this analytic sample occurred June 2009 through May 31, 2011, and the study participation rate was 71%. As of May 31, 2011, 1042 women had enrolled in the study. Furthermore, after geocoding the data, we restricted this analysis to the 693 women whose childhood residential history was geocoded to an acceptable address (as described further below). This research study was approved by the Providence Hospital and Medical Centers Institutional Review Board (IRB), the Wayne State University IRB Behavioral (B3) Committee, and the Northeastern University IRB. These data (entitled: LIFE Replication Dataset, Detroit, MI (2009–2011)) are available as a replication dataset from openICPSR at University of Michigan, DOI # 10.3886/E31393V1, Principal Investigator: Dawn Misra. They can be accessed via: http://doi.org/10.3886/E31393V1

### Childhood Subjective Neighborhood Measures

Women answered retrospective survey questions for two subjective neighborhood scales, and a one-item violence victimization measure, all referring to the neighborhood context in which the woman lived in childhood at her 10^th^ birthday. The measures were adapted from valid, reliable scales originally designed to refer to the current (adulthood) neighborhood context. [11–15] Missing data was small (<3% of observations imputed to the nonmissing mean of each item (or to the mode, for neighborhood victimization)).

To measure childhood neighborhood physical and social disorder, participants reported on 6 items using a 3-point likert scale how much of a problem each item was in her childhood neighborhood including: (1) litter/ trash on sidewalks/streets; (2) graffiti; (3) vacant /deserted houses/storefronts; (4) public drinking; (5) selling/using drugs; (6) groups hanging out and causing trouble.[13–15] To measure childhood neighborhood informal social control, participants reported on 4 items with a 5-point likert scale how likely that people from her childhood neighborhood would do something to intervene: (1) If children were skipping school and hanging out on a street corner; (2) If children were spray-painting graffiti; (3) If a child was showing disrespect to an adult; (4) If there was a fight in front of her house and someone was being beaten/ threatened. [11–14] We reverse coded relevant items and summed the scales; higher scale values indicate higher disorder or higher social control.

The measure for past neighborhood violent victimization asked women about whether anyone ever use violence against her or a household member when she lived in her childhood neighborhood, coded as 1 if yes and 0 otherwise.

### Adult Subjective Neighborhood Measures

For one of our analyses, women answered survey questions on neighborhood physical and social disorder and neighborhood violent victimization referring to their current neighborhood context. The same items, measures, and methods for measuring neighborhood social disorder and victimization at age 10 (described above) were used to measure current neighborhood context.

### Residential History and Geocoding Methods

Each woman was asked to report the residential address where she lived when she was aged 10, as well as her current residential address, how long she had lived at her current (adult) address in months (residential stability), and her current adult age. If she could not recall the exact addresses, she was asked to provide the nearest cross-streets, or landmarks. Addresses were then geocoded to latitude and longitude coordinates using the ArcGIS Online US Streets 10.0 geocoding service, as well as using the Google Maps geocoding service via the GPS Visualizer interface.[16] Addresses were matched to varying degrees of precision; acceptable precision for geocoding included addresses geocoded to the street address, on the building at the street address, or at the nearest intersection. Of the 1042 women enrolled in the LIFE study on 5/31/11, 13% (n = 138) provided insufficient information to geocode their childhood address; of the remaining addresses, 211 (20%) were unacceptable geocodes, and 693 (67%) were acceptable. For current addresses, 1% (n = 14) provided insufficient information for geocoding, 93 (9%) addresses resulted in unacceptable geocodes, and 935 (90%) were acceptable.

### Objective Neighborhood Data

Each residential address (using its latitude/longitude coordinates) was then geocoded to a census tract identifier. Childhood addresses were geocoded using Census 2000 geography, and were merged to 1970–2000 census tract data where tract boundaries were normalized over time by the Geolytics company [17] to those from 2000. This normalization method therefore recreated all tracts from 1970–1990 to have the same boundaries as tracts from 2000, to control for artifactual causes of neighborhood changes across time due to regrouping of dissimilar population compositions. We linearly interpolated the census variable values between decades, and assigned an (interpolated) census tract value based on the year of the woman’s 10^th^ birthday (derived from her birthdate). For the women whose 10^th^ birthday occurred after year 2000 (n = 33), we applied 2000 values. We geocoded the current address in adulthood to Census 2000 geography. We analyzed 3 objective (census derived) variables: tract % black, tract % poverty, and a derived standardized tract deprivation score (where higher values indicate worse deprivation). The neighborhood deprivation variable is a factor score derived from the first factor extracted from a nation-wide exploratory principal components analysis (PCA)[18] of 5 tract variables: % poverty, % on welfare or public assistance, % of those aged 16+ unemployed, % female headed households with children, and % of those aged 25+ with less than a high school education. PCA methods were used to operationalize the underlying construct of neighborhood deprivation because they maximize the measure reliability, reduce measurement error, and are robust to problems inherent to any single variable. [19] Theoretically, compilation measures emphasize the accumulation of disadvantage, and are conceptually appealing since households use multiple criteria to select a neighborhood, not just one. Such measures are being increasingly utilized by housing agencies and advocates to guide translation, including to target neighborhoods for affordable housing development or for neighborhood revitalization.[20] Tract variable selection was guided by neighborhood concentrated disadvantage literature. [11,12,14,19,21] We confirmed high internal consistency reliability for each decade of data (Cronbach’s alpha range: .89–.92). Standardized scoring coefficients were used to construct the factor scores, and each of the 5 variables contributed approximately equal weight; the first factor explained over 64% of the variance in the data in each decade (range: 65%-75%).

### Adult Health Measures

We tested for recall bias of the subjective neighborhood measures (described below), by three common measures of adulthood (current) health: depressive symptomatology, self-rated health, and low birthweight. Depressive symptomatology was measured using the 20-item Center for Epidemiological Studies Depression (CES-D) Scale. Women reported how frequently they experienced depressive symptoms during the past week based on a 4-point likert scale: 1 = rarely or none of the time, to 4 = most or all of the time. The CES-D scale includes items such as “I was bothered by things that usually don’t bother me,” and “I felt that everything I did was an effort.” Items reflecting positive feelings were reverse coded. Scale items were summed; higher scale values indicate more depressive symptoms. The few missing values for CES-D scale items were imputed to the item-specific mean of the sample (<1% missing per item).

Women self-reported their current physical health based on a 5-point likert scale: 1 = excellent, 2 = very good, 3 = good, 4 = fair, and 5 = poor. Responses were reverse coded; higher self-rated health (SRH) scores indicate better perceived physical health. The few missing values for self-rated physical health were imputed to the sample mean (<1% missing). Low birthweight (LBW) was defined as giving birth to an infant that was < 2,500 grams at birth, compared to weight above this threshold as the reference, abstracted from the medical record. The one observation missing a birthweight measure was imputed to the mode.

### Analytic Methods

We tested internal consistency reliability of the retrospective subjective childhood neighborhood scales, as well as of the subjective current neighborhood scales, by calculating standardized Cronbach’s alpha. Construct validity is defined as whether the measured variable accurately represents the higher order construct,[22] and whether the measure behaves the way it should behave in relation to other constructs.[23] We tested construct validity of the childhood subjective neighborhood measures by calculating correlations (1) among each of those measures (“the subjective neighborhood measures”) with the 3 census-based objective childhood neighborhood measures (“the objective neighborhood measures”), (2) among the 3 subjective childhood neighborhood measures, and (3) among the 3 objective childhood neighborhood measures. We used Pearson correlations for the objective measures and nonparametric Spearman correlations to test associations with the subjective neighborhood measures, given that neighborhood victimization was a binary measure, although results were similar with Pearson tests. We also tested validity of the neighborhood victimization measure by conducting t-tests with the continuous neighborhood variables.

We tested reliability and validity of the subjective neighborhood measures by comparing women’s subjective neighborhood reports at the two time points of childhood and adulthood (currently). In addition to assessing the entire sample to test this hypothesis, we separately tested the reliability and validity for a subgroup of women in our sample who were residentially stable at their current adult address (defined at the sample mean, more than 57.9 months), and for different subsets of women who lived at the same address or neighborhood during both childhood and adulthood. We hypothesized that the correlations between current (adult) and childhood subjective neighborhood context would be positive, and stronger when the sample was restricted to women who (1) were residentially stable at their current adult address, (2) lived in the same address at both times, (3) lived in the same census tract at both times, and (4) lived in the same tract at both times while the objective neighborhood remained similar according to the census deprivation measure. For this sub-analysis (4), we operationalized a “similar tract” at both time points by calculating the difference between the current and age 10 tract deprivation values, then restricted the subsample to those difference values falling within a half standard deviation of zero (plus or minus .5 of 1 SD), which allowed for some degree of error in the difference measure. We tested neighborhood disorder concordance across time with spearman correlations, and neighborhood victimization across time, with tetrachoric correlations and kappa tests in the full sample and in these four subsamples.

We formally tested for potential recall bias, to investigate whether participants exhibiting worse adult health also reported subjectively worse (or better) childhood neighborhood context, compared to the quality of the neighborhood as indicated by objective Census data. Using linear regression, we regressed each of the subjective childhood neighborhood context measures on the independent predictors of one measure of objective childhood neighborhood context, and one health measure (i.e., depressive symptoms, SRH, or LBW), and their interaction. We then repeated for all combinations of subjective neighborhood, objective neighborhood, and health measures. A significant interaction (2-sided test, alpha: p<.05) between health and objective childhood context would provide evidence for recall bias, since it suggests that participants in poorer health reported a significantly worse (or better) childhood subjective environment than the objective childhood neighborhood measure suggests.

We lastly conducted several sensitivity analyses that we report as Supplemental Information, including to test whether the observations that could not be geocoded were similar to those that were geocoded; to test for recall bias of the subjective childhood neighborhood measures, by age and residential stability of the adult (current) address; and to compare the residential stability in our sample to the US population to inform generalizability.

## Results

### 1. Descriptive Analysis

The postpartum women in the LIFE sample had a mean age of 27.5 (SD = 6.20), range of 18–45 (Table 1). Their mean birth year was 1982; so the year of the women’s 10^th^ birthday occurred 10 years later (range: 1976 to 2002, mean of 1992).

**Table 1:** LIFE Study Neighborhood at Age 10 and Current Health Univariates.

	Mean or N	Std Dev or %	Min	Max
Enrollment Age	27.54	6.2	18	45
Childhood City of Residence (N; %)				
Detroit	567	82%		
Southfield	33	5%		
Oak Park	19	3%		
Other Michigan Places	50	7%		
Outside Michigan	24	3%		
Childhood Neighborhood of Residence				
Neighborhood Informal Social Control Scale	16.23	4.15	4	20
Neighborhood Physical & Social Disorder Scale	9.22	3.73	6	18
Neighborhood Victimization (N; %)	119	17%		
Objective Neighborhood Measures				
Tract % Poverty	0.23	0.13	0	0.69
Tract % Black	0.79	0.26	0	1
Tract Deprivation Factor Score	1.44	1.22	-1.23	5.05
Residential Stability at Current Address (months)	57.90	86.01	0	504
Current Health Indicators				
CES-D Score	15.59	9.74	0	53
Self-Rated Physical Health	2.76	1.08	1	5
Low Birthweight (N; %)	94	14%		

N = 693

Based on the geocoded address that each woman recalled for her 10^th^ birthday, 82% of the sample reported living in the city of Detroit, with the remaining majority living in another Michigan town. Four percent lived outside Michigan.

### 2. Comparing the Geocoded to the Nongeocoded Samples

Across the vast majority of sample characteristics, those with and without a successful childhood address geocode were comparable (See Supporting Information, Table A in S1 File). However those that could not be geocoded were more likely to have missing current income, and were less likely to live in Detroit city (compared to outside the city). Although one significant test emerged suggesting differences by residential stability at the current address, this result was driven by influential outliers. Tests were nonsignificant by geocoding status when excluding outliers or using nonparametric measures or proportions to operationalize residential stability.

### 3. Scale Internal Consistency Reliability

The internal consistency reliability of the retrospective subjective scale measures of both childhood and adult context was high. Childhood neighborhood disorder: Cronbach’s α = .93, childhood neighborhood informal social control: α = .89, and adult (current) neighborhood disorder: Cronbach’s α = .92. Deletion of none of the items raised the reliability.

### 4. Construct Validity

#### 4a. Subjective and Objective Childhood Neighborhood Correlations

For our main construct validity analysis, we documented moderate Spearman correlations for neighborhood disorder, moderate to weak correlations for neighborhood informal social control and neighborhood victimization, each with census-based objective measures of childhood neighborhood context. All correlations were in the expected direction (Table 2). Subjective-objective neighborhood associations were over 2 times stronger with neighborhood disorder than with neighborhood informal social control or with neighborhood victimization, with strongest correlations for subjective neighborhood disorder with the objective measures of neighborhood deprivation and neighborhood poverty (rhos of .40, p<.001). Correlations were moderate to weak among neighborhood disorder with neighborhood % black (rho = .26, p<.001). However the absolute value of the correlations with neighborhood social control and neighborhood victimization were all weak, at rhos of less than .20.

**Table 2:** Objective-Subjective Childhood (age 10) Neighborhood Correlations at Age 10, LIFE Study.

	Spearman Correlations					
	Neighborhood Deprivation		Neighborhood Poverty		Neighborhood Share Black	
	Rho	p	Rho	p	Rho	p
Childhood neighborhood social disorder	0.40	***	0.40	***	0.26	***
Childhood neighborhood social control	-0.14	***	-0.16	***	-0.08	*
Childhood neighborhood victimization	0.17	***	0.17	***	0.10	**

*** p<.001

** p<.01

*p<.05

#p<.10 n = 693.

#### 4b. Childhood Subjective and Objective Neighborhood: T-tests

As reported in Table 3, we found significantly worse neighborhood quality for women who reported any (as opposed to no) neighborhood victimization, as hypothesized, including lower neighborhood social control, higher neighborhood disorder, neighborhood deprivation, neighborhood poverty, and neighborhood % black (all at least p<.02).

**Table 3:** T-Tests of Difference in Childhood Neighborhood Context by Childhood Neighborhood Victimization; LIFE Study.

	No Childhood Neighborhood Victimization			Yes Childhood Neighborhood Victimization			Difference in Means
Variable	Mean	95% LCL	95% UCL	Mean	95% LCL	95% UCL	P
Childhood Neigh Social Control	16.72	(16.41,	17.03)	13.83	(12.92,	14.73)	<.0001
Childhood Neigh Disorder	8.62	(8.34,	8.89)	12.12	(11.35,	12.88)	<.0001
Childhood Tract Deprivation Factor	1.35	(1.25,	1.44)	1.91	(1.68,	2.15)	<.0001
Childhood Tract % Poverty	0.219	(0.209,	0.230)	0.282	(0.257,	0.307)	<.0001
Childhood Tract % Black	0.777	(0.754,	0.799)	0.835	(0.794,	0.875)	0.014

N = 693. Neigh = neighborhood.

#### 4c. Subjective childhood neighborhood correlations

The spearman correlation between neighborhood disorder and social control was moderate and in the expected (inverse) direction (rho = -.53, p<.0001). Correlations with childhood neighborhood victimization were lower but moderate and statistically significant in the expected directions; rho = .32 (p<.0001) for neighborhood disorder, and rho = -.23 (p<.0001) for neighborhood informal social control.

#### 4d. Objective childhood neighborhood correlations

As hypothesized, the Pearson correlation was strong (rho = .94, p<.0001) between tract poverty and tract deprivation. There were moderate correlations between tract % black and tract deprivation (rho = .50, p<.0001), and between tract % black and tract % poverty (rho = .35, p<.0001).

### 5. Adulthood compared to childhood neighborhood context

Among all women, the correlation between neighborhood disorder in childhood and in adulthood was small (rho = .05, p = .19) (Table 4). Correlations were stronger when the sample was restricted to subsets of the sample with higher residential stability for their current (adulthood) address. For example, among women who were above the sample mean for residential stability for their current address, the childhood-adulthood neighborhood disorder correlation was .13. Among women who lived in the same neighborhood at both times, who lived in the same neighborhood with similar objective neighborhood conditions at both times, or who lived at the same address at both times, the correlations for neighborhood disorder rose, up to .48 (p<.01).

**Table 4:** Comparing childhood and adulthood neighborhood measures, entire sample and among residentially-stable subgroups; LIFE Study.

			Respondents who lived in the same neighborhood and/or same address between age 10 and adulthood		
	Entire sample	Residen-tially stable ^c^	Same neighborhood ^d^	Same neighborhood & similar neighborhood deprivation ^e^	Same address
Neighborhood Disorder					
Rho^a^	0.050	0.129	0.475	0.484	0.438
p	0.190	0.0801	<.0001	<.0001	0.008
N	680	184	84	73	36
Neighborhood victimization					
Rho^b^	0.372	0.460	0.467	0.510	0.722
p	<.0001	0.0021	0.0235	0.0138	0.002
N	689	186	85	74	36
Neighborhood victimization					
Kappa	0.141	0.229	0.246	0.279	0.438
p	<.0001	0.0016	0.023	0.016	0.004
N	689	186	85	74	36

NOTES:

^a^ Pearson correlation;

^b^ Tetrachoric correlation;

^c^ Residentially Stable defined as Length of Residence at Current (adult) Address above the sample mean of 57.9 months;

^d^ neighborhood is operationalized as the census tract;

^e^ similar level of neighborhood deprivation is operationalized as a comparable level of the deprivation factor score at both timepoints, within a standard deviation of the mean difference.

The tetrachoric correlation between the childhood and adulthood (binary) neighborhood victimization variable was rho = .37 for the entire sample, and considerably higher for those with higher residential stability, living in the same neighborhood, in similar neighborhoods at both times, or at the same address (all p<.05, Table 4). Agreement of neighborhood victimization between childhood and adulthood periods was low for the entire sample (kappa = .14, p<.001), but rose considerably when restricting to participants who were residentially stable (kappa = .23), to those living in the same neighborhood or type of neighborhood at both times (kappa range .25–.28, p<.03, Table 4), or living at the same address at both time points (kappa = .44, p = .004).

### 6. Recall Bias of Subjective Childhood Neighborhood Measures by Adult Health Status

Table 5 presents the interaction coefficients from linear regression models, where each subjective measure of childhood neighborhood context was regressed on one objective childhood neighborhood measure, one adult health indicator, and their interaction. We found limited evidence that the measures of subjective childhood neighborhood context exhibited recall bias by current adult health. We did find that women who gave birth to a low birthweight child may have differentially reported neighborhood social control in childhood. However there were few interactions achieving statistical significance otherwise for the other health measures, or other neighborhood measures.

**Table 5:** Recall Bias Analysis: Interactions between Health Indicators and Objective Childhood Neighborhood Measures Predicting Subjective Childhood Neighborhood Context; LIFE Study.

	Childhood Neighborhood Social Control			Childhood Neighborhood Social Disorder			Childhood Neighborhood Victimization		
Interaction	β	SE	P	β	SE	P	β	SE	P
CES-D Score									
X Past Neigh Deprivation	0.009	0.013	0.463	0.004	0.011	0.732	-0.008	0.008	0.323
X Past Neigh Poverty Rate	0.100	0.118	0.399	0.076	0.100	0.448	-0.046	0.070	0.516
X Past Neigh Share Black	0.016	0.060	0.790	-0.065	0.053	0.220	0.024	0.042	0.567
Self-Rated Physical Health									
X Past Neigh Deprivation	-0.009	0.118	0.939	0.039	0.101	0.698	0.067	0.081	0.404
X Past Neigh Poverty Rate	-0.138	1.104	0.900	0.275	0.939	0.770	0.529	0.727	0.466
X Past Neigh Share Black	0.486	0.509	0.341	0.090	0.450	0.841	0.206	0.383	0.590
Low Birthweight									
X Past Neigh Deprivation	1.120	0.336	0.001	-0.419	0.288	0.146	-0.050	0.031	0.108
X Past Neigh Poverty Rate	9.821	2.991	0.001	-3.131	2.562	0.222	-0.380	0.275	0.168
X Past Neigh Share Black	3.531	1.510	0.020	-2.237	1.341	0.096	-0.279	0.138	0.043

N = 693 for most models. N = 686 for neighborhood deprivation models. Note: Childhood neighborhood operationalized as neighborhood at age 10. “Neigh” = neighborhood. Each health measure, and each objective neighborhood measure, was tested in a separate model, totaling 27 models altogether.

### 7. Recall Bias of Subjective Childhood Neighborhood Measures by Age and Residential Stability

Table B in S1 File presents results testing for recall bias by age and residential stability of the adulthood current address. We found only 1 significant interaction test, between residential stability and neighborhood poverty on victimization. Given that we ran 18 tests here, we would expect to see at least one significant effect by chance. Therefore, neither age nor residential stability seem to be generating recall bias of our subjective childhood measures.

## Discussion

Although exposures in childhood are increasingly being documented as predictors of adult health outcomes, [5,24,25] the operationalization of childhood exposures remains crude. Although parental SES is frequently used and indeed predicts adult health independently of adult SES, [26,27] other aspects of childhood context, including those patterned by one’s neighborhood, are likely important, and may be even more distal predictors of parental SES.[28,29] Although most neighborhood health studies have enrolled adults, inequality is imprinted on development starting early in life. [8,30] To advance knowledge on the importance of childhood exposures for health throughout life, we argue that the literature must measure early life place-related context. Given the high magnitude of neighborhood inequality and racial residential segregation and their impact on other forms of life course inequality in the US, [31,32] modeling effects of neighborhood context early in life might be especially important for understanding adult health and racial health disparities in the US.

Our results provide evidence that retrospective reports of subjective neighborhood context in childhood are a valid, reliable method to operationalize early life neighborhood context for epidemiologic studies. Some constructs such as one’s own anthropometry at birth and some measures of parental SEP are relatively accurately reported retrospectively, [33–35] although they may be recalled with error, therefore associations with health may be biased towards the null. Retrospective reports are more efficient than measures from prospective cohort studies begun in childhood, and are able to provide more nuanced measurement than administrative record linkage. Indeed, subjective neighborhood reporting will generate more complete data than collection of address histories, since even when respondents may not recall their exact addresses accurately, they may still provide a subjective report of that neighborhood.

Although influential birth cohort studies have been established in Europe, [36,37] most cohort studies in the US have enrolled adults, and older adults at that. Although some US prospective cohort studies have begun in childhood, span a long period of time, and have a focus on health (Wisconsin Longitudinal Study), many have only limited health measures, or the current age for adult disease follow up remains relatively young (Add Health, National Longitudinal Study of Youth; Early Childhood Longitudinal Study; Panel Study of Income Dynamics; National Children’s Study). Therefore the utility of US based cohort studies to measure childhood neighborhood context prospectively for adult health associations is limited.

We operationalized childhood address here by the address at the woman’s 10^th^ birthday, in middle childhood, in line with other studies that have used a measure at one point in time to represent childhood constructs such as SES. [38,39] However there may be more measurement error for families that moved in childhood. Other studies have documented that within residential histories, moves across time are to similar types of neighborhoods.[40] Therefore, while there may be some measurement error on the exact address that women report, the characteristics associated with those addresses may be similar.

We found that the retrospective subjective measure of neighborhood physical and social disorder in childhood was moderately correlated with objective (census based) characteristics of the childhood context, which is in line with the wide range of correlations documented in other studies for various measures of adult neighborhood only. [12,41] For example, in the Project on Human Development in Chicago Neighborhoods study, correlations between neighborhood concentrated disadvantage and neighborhood social constructs (such as reciprocated exchange) in adults ranged from-.27 to-.55.[12] We might expect our neighborhood associations to be weaker, given they reference past context, and may contain more measurement error.

The neighborhood environment has been documented as influencing a range of other (more proximal) social and environmental determinants of health, including socioeconomic position, school quality, housing quality, social networks, violence, noise, drug markets, environmental exposures (e.g. brownfields, or air pollution), healthy foods, and advertising of harmful products like alcohol and tobacco. [42,43] All of these may be important exposures in childhood, associated with causes of later life disease, and therefore related to one’s childhood residential environment. In addition to being a distal cause of disease, neighborhood environment in childhood also represents a confounder, as a prior cause of adult neighborhood of residence, of adult socioeconomic position, and of other behaviors that are associated with adult risk of chronic disease. It is unclear how influential the omission of prior neighborhood context may be for health studies because so few studies have included it. [8] This is an important direction for future research.

### Limitations

We found that subjective and objective neighborhood measures were only moderately correlated. Although this aligns with prior research, [12,41] it also suggests that each captures something unique about the neighborhood. First, the areas that people consider their “neighborhood” are considerably heterogeneous between people, and may even differ based on the purpose of defining the neighborhood. [44] While the areas considered for census tracts have been constructed to be relatively homogenous, they encompass rather large areas (population means of 4,000). Subjective measures may capture a smaller area. Moreover, subjective measures capture the social environment which is difficult to operationalize with administrative data, and may mediate structural neighborhood characteristics on other outcomes including health.[12]

Since subjective measures are self-reported in adulthood, they could also exhibit reporting bias, since retrospective measures may be particularly subject to recall bias.[45] However, we found minimal evidence that our subjective measures of childhood neighborhood exhibited recall bias by current (adult) health, by respondent’s current age, or by residential stability of current address. We did find that one measure, of childhood neighborhood informal social control, may exhibit recall bias for mothers of low birthweight babies, although we observed this bias consistently for only this one subjective measure, not for the other two subjective childhood neighborhood measures, or for the other health outcomes. It is possible that the measures may still exhibit recall bias by other characteristics beyond which we tested in this manuscript, if, for example, some residents are conditioned over time to become desensitized or more sensitized [46] to adverse neighborhoods, or if some residents remembered the past differently (e.g. romanticizing it), or if certain places were more meaningful to people than others.[47] A related theoretical issue of measurement concerns how children, versus how adults, perceive and report the quality of their neighborhoods, which could differ, although some prior literature has documented that the two perceptions are positively correlated, and correlated with objective neighborhood context. [48]

Our sample was comprised of black women of childbearing age who gave birth in a Detroit area hospital. We pursued this recruitment strategy for several reasons related to our main study aims which are focused on understanding the higher risk of adverse birth outcomes among black women. Since women rarely give birth outside of a hospital in the US, community sampling is inefficient for understanding birth outcomes, and vital statistics data would not provide the detail we required to test our study aims. We chose to recruit at Providence Hospital in Southfield Michigan because it has a wide catchment area, and it generates a high volume of annual births, including a high volume of births to black women of heterogeneous backgrounds. Therefore enrollment through this hospital enabled us to generate a sample of heterogeneous black women, which we believe is representative of a range of black women of childbearing age in this region.

To test whether results for measuring childhood context from our sample might be generalizable to broader populations, we compared adulthood residential stability in our sample to that of the US population, and of population subgroups matching our sample, from the US Census Bureau [49]. Our sample was more residentially mobile than either the US population as a whole, those aged 18–44, or Blacks 18–44 (See Table C in S1 File). If residential mobility in adulthood is correlated with residential mobility in childhood, and if residential mobility in childhood causes measurement error (including potentially worse recall in retrospective measures), thereby lowering validity and reliability, we would expect that the subjective measures of childhood neighborhood context would exhibit even stronger psychometric properties in more residentially stable populations. Indeed, we then tested this hypothesis explicitly (see Table 4), and found that, in the subset of our sample with higher residential stability, the psychometric properties of the subjective measure are stronger. The results from our study therefore represent a conservative estimate compared to those from more stable populations.

Our analysis relied on a subset of data for whom we had measured both subjective and objective neighborhood context in childhood, and we had to exclude those with childhood addresses that could not be geocoded (a third of the sample). As reported in the results, those with and without a successful childhood address geocode were comparable across most sample characteristics. However it is possible that those with a valid geocode were more residentially stable in childhood (and therefore better able to recall their age-10 address). However we cannot formally test residential mobility in childhood per se with our data. We did rule out that recall bias by residential mobility is a predominant explanation for the subjective measures in our data. However, as with the discussion above about generalizability, we can speculate that including the excluded participants who had been more residentially mobile would likely have introduced more measurement error into our analysis. Obtaining precise addresses in a residential history going back to childhood is challenging and this is one reason we introduce this subjective neighborhood measure, to avoid having to exclude participants who cannot recall the exact address, but can instead recall the type of neighborhood in which they lived.

In conclusion, we found that retrospective subjective childhood neighborhood measures exhibited good validity and reliability. Despite the limitations of our approach, this study is a necessary first effort to address a key gap in the literature to explore the measurement properties and of the childhood residential environment. Capturing neighborhood context by survey, whether concurrently or retrospectively, is efficient as well as a promising novel approach for incorporating the simultaneous context, as situated in both place and time, into our understanding of what causes health and disease across the life course.

## Supporting Information

S1 File Supporting Information Tables for the LIFE Measures of Childhood Neighborhood Context. (DOCX)

## References

[1] Chaix B (2009) Geographic Life Environments and Coronary Heart Disease: A Literature Review, Theoretical Contributions, Methodological Updates, and a Research Agenda. Annu Rev Public Health 30: 81–105. 10.1146/annurev.publhealth.031308.100158 19705556

[2] Mair C, Diez Roux AV, Galea S (2008) Are neighbourhood characteristics associated with depressive symptoms? A review of evidence. J Epidemiol Community Health 62: 940–946. 10.1136/jech.2007.066605 18775943

[3] Yen IH, Michael YL, Perdue L (2009) Neighborhood Environment in Studies of Health of Older Adults: A Systematic Review. American Journal of Preventive Medicine 37: 455–463. 10.1016/j.amepre.2009.06.022 19840702 PMC2785463

[4] Diez Roux AV, Mair C (2010) Neighborhoods and health. Annals of the New York Academy of Sciences 1186: 125–145. 10.1111/j.1749-6632.2009.05333.x 20201871

[5] Lynch J, Davey Smith G (2005) A Life Course Approach To Chronic Disease Epidemiology. Annual Review of Public Health 26: 1–35. 15760279 10.1146/annurev.publhealth.26.021304.144505

[6] Kuh D, Ben Shlomo Y, editors (2004) A Life Course Approach to Chronic Diseases Epidemiology 2nd Edition ed: Oxford University Press.

[7] Kuh D, Ben-Shlomo Y, Lynch J, Hallqvist J, Power C (2003) Glossary: Life course epidemiology. J Epidemiol Community Health 57: 778–783. 14573579 10.1136/jech.57.10.778 PMC1732305

[8] Osypuk TL (2013) Future research directions for understanding neighborhood contributions to health disparities. Revue d'Epidemiologie et de Sante Publique 61: S61–S68. 10.1016/j.respe.2013.03.040 23660539 PMC3714396

[9] Cerda M, Diez-Roux AV, Tchetgen ET, Gordon-Larsen P, Kiefe C (2010) The Relationship Between Neighborhood Poverty and Alcohol Use: Estimation by Marginal Structural Models. Epidemiology 21: 482–489. 10.1097/EDE.0b013e3181e13539 20498603 PMC3897210

[10] Nandi A, Glass TA, Cole SR, Chu H, Galea S, Celentano DD, et al (2010) Neighborhood Poverty and Injection Cessation in a Sample of Injection Drug Users. American Journal of Epidemiology 171: 391–398. 10.1093/aje/kwp416 20093307 PMC2877451

[11] Sampson R, Raudenbush S, Earls F (1997) Neighborhoods and violent crime: a multilevel study of collective efficacy. Science 277: 918–924. 9252316 10.1126/science.277.5328.918

[12] Sampson R, Morenoff JD, Earls F (1999) Beyond social capital: spatial dynamics of collective efficacy for children. American Sociological Review 64: 633–660.

[13] Earls FJ, Brooks-Gunn J, Raudenbush SW, Sampson RJ (1994) Project on Human Development in Chicago Neighborhoods: Community Survey, 1994–1995 Data Collection Instrument. ICPSR 2766. Ann Arbor, MI: ICPSR, University of Michigan.

[14] Sampson RJ, Raudenbush SW (1999) Systematic Social Observation of Public Spaces: A New Look at Disorder in Urban Neighborhoods. American Journal of Sociology 105: 603–651.

[15] Sampson R, Raudenbush S (2004) Seeing Disorder: Neighborhood Stigma and the Social Construction of "Broken Windows". Social Psychology Quarterly 67: 319–342.

[16] Schneider A (2012) GPS Visualizer http://www.gpsvisualizer.com/geocoder/.

[17] Geolytics (2003) Neighborhood Change Database Longform Release 2.0. East Brunswick, NJ.

[18] Hatcher L (1994) Chapter 1: Principal Component Analysis A Step-by-Step Approach to Using SAS for Factor Analysis and Structural Equation Modeling. Cary, NC: SAS Institute Inc.

[19] Messer LC, Laraia BA, Kaufman JS, Eyster J, Holzman C, Culhane J, et al (2006) The development of a standardized neighborhood deprivation index. Journal of Urban Health 83: 1041–1062. 17031568 10.1007/s11524-006-9094-x PMC3261293

[20] Osypuk TL (2015) Shifting from policy relevance to policy translation: Do housing and neighborhoods affect children’s mental health? Social Psychiatry and Psychiatric Epidemiology 50: 215–217. 10.1007/s00127-014-0998-6 25527210 PMC5082417

[21] Merolla DM, Hunt MO, Serpe RT (2011) Concentrated Disadvantage and Beliefs about the Causes of Poverty: A Multi-Level Analysis. Sociological Perspectives 54: 205–228.

[22] Shadish WR, Cook TD, Campbell DT (2002) Experimental and Quasi-experimental Designs for Generalized Causal Inference. New York: Houghton Mifflin Co.

[23] DeVellis RF (1991) Scale Development: Theory and Applications. Newbury Park, CA: Sage Publications.

[24] Barker D (1995) Fetal origins of coronary heart disease. BMJ 311: 171–174. 7613432 10.1136/bmj.311.6998.171 PMC2550226

[25] Campbell F, Conti G, Heckman JJ, Moon SH, Pinto R, Pungello E, et al (2014) Early Childhood Investments Substantially Boost Adult Health. Science 343: 1478–1485. 10.1126/science.1248429 24675955 PMC4028126

[26] Galobardes B, Lynch JW, Davey Smith G (2004) Childhood Socioeconomic Circumstances and Cause-specific Mortality in Adulthood: Systematic Review and Interpretation. Epidemiologic Reviews 26: 7–21. 15234944 10.1093/epirev/mxh008

[27] Galobardes B, Smith GD, Lynch JW (2006) Systematic Review of the Influence of Childhood Socioeconomic Circumstances on Risk for Cardiovascular Disease in Adulthood. Annals of Epidemiology 16: 91–104. 16257232 10.1016/j.annepidem.2005.06.053

[28] Cutler DM, Glaeser EL (1997) Are Ghettos Good or Bad? Quarterly Journal of Economics 112: 827–872.

[29] Glymour MM, Avendano MP, Berkman LF (2007) Is the stroke belt worn from childhood? Risk of first stroke and state of residence in childhood and adulthood. Stroke 38: 2415–2421. 17673716 10.1161/STROKEAHA.107.482059

[30] Hertzman C, Boyce T (2010) How experience gets under the skin to create gradients in developmental health. Annu Rev Public Health 31: 329–347. 10.1146/annurev.publhealth.012809.103538 20070189

[31] Acevedo-Garcia D, Rosenfeld LE, Hardy E, McArdle N, Osypuk TL (2013) Future Directions in Research on Institutional and Interpersonal Discrimination and Child Health. American Journal of Public Health 103: 1754–1763. 10.2105/AJPH.2012.300986 23409880 PMC3780723

[32] Acevedo-Garcia D, Osypuk TL, McArdle N, Williams D (2008) Toward a Policy-Relevant Analysis of Geographic and Racial/Ethnic Disparities in Child Health. Health Affairs 27: 321–333. 10.1377/hlthaff.27.2.321 18332486

[33] Straughen JK, Caldwell CH, Osypuk TL, Helmkamp L, Misra DP (2013) Direct and Proxy Recall of Childhood Socio-Economic Position and Health. Paediatric and Perinatal Epidemiology 27: 294–302. 10.1111/ppe.12045 23574418 PMC3637926

[34] Walton KA, Murray LJ, Gallagher AM, Cran GW, Savage MJ, Boreham C (2000) Parental recall of birthweight: A good proxy for recorded birthweight? European Journal of Epidemiology 16: 793–796. 11297220 10.1023/a:1007625030509

[35] Batty GD, Lawlor DA, Macintyre S, Clark H, Leon DA (2005) Accuracy of adults’ recall of childhood social class: findings from the Aberdeen children of the 1950s study. Journal of Epidemiology and Community Health 59: 898–903. 16166367 10.1136/jech.2004.030932 PMC1732932

[36] Lucas P, Allnock D, Jessiman T (2013) How are European birth-cohort studies engaging and consulting with young cohort members? BMC Medical Research Methodology 13: 56 10.1186/1471-2288-13-56 23578172 PMC3651350

[37] Birthcohorts.net (2013) http://www.birthcohorts.net/.

[38] Carson AP, Rose KM, Catellier DJ, Kaufman JS, Wyatt SB, Diez–Roux AV, et al (2007) Cumulative Socioeconomic Status Across the Life Course and Subclinical Atherosclerosis. Annals of Epidemiology 17: 296–303. 17027292 10.1016/j.annepidem.2006.07.009

[39] Pollitt RA, Kaufman JS, Rose KM, Diez-Roux AV, Zeng D, Heiss G (2008) Cumulative life course and adult socioeconomic status and markers of inflammation in adulthood. Journal of Epidemiology and Community Health 62: 484–491. 10.1136/jech.2006.054106 18477746

[40] Murray ET, Diez Roux AV, Carnethon M, Lutsey PL, Ni H, O’Meara ES (2010) Trajectories of Neighborhood Poverty and Associations With Subclinical Atherosclerosis and Associated Risk Factors: The Multi-Ethnic Study of Atherosclerosis. American Journal of Epidemiology 171: 1099–1108. 10.1093/aje/kwq044 20423931 PMC2877469

[41] Echeverria SE, Diez-Roux AV, Link BG (2004) Reliability of self-reported neighborhood characteristics. Journal of Urban Health 81: 682–701. 15466849 10.1093/jurban/jth151 PMC3455923

[42] Kawachi I, Berkman LF (2003) Neighborhoods and Health. New York, NY: Oxford University Press.

[43] Acevedo-Garcia D, Osypuk TL (2008) Impacts of Housing and Neighborhoods on Health: Pathways, Racial/Ethnic Disparities, and Policy Directions In: Carr JH, Kutty NK, editors. Segregation: The Rising Costs for America. New York: Routledge pp. 197–235.

[44] O'Campo P (2003) Invited commentary: Advancing theory and methods for multilevel models of residential neighborhoods and health. American Journal of Epidemiology 157: 9–13. 12505885 10.1093/aje/kwf171

[45] Szklo M, Nieto FJ (2000) Epidemiology: Beyond the Basics. Gaithersburg, MD: Aspen Publishers, Inc.

[46] Rutter M (2012) Resilience as a dynamic concept. Development and Psychopathology 24: 335–344. 10.1017/S0954579412000028 22559117

[47] Dunn JR (2002) A Population Health Approach to Housing: A Framework for Research. Ottawa.

[48] Farver JAM, Ghosh C, Garcia C (2000) Children's Perceptions of Their Neighborhoods. Journal of Applied Developmental Psychology 21: 139–163.

[49] US Census Bureau, Annual Social and Economic Supplement (ASEC) to the Current Population Survey (CPS) (2010) Current Population Survey Data on Migration/Geographic Mobility, 2005–2010 and 2009–2010. Detailed Tables. http://www.census.gov/hhes/migration/data/cps.html. Washington, DC: US Census Bureau.

