# Supplementary material for: Where We Used to Live: Validating Retrospective Measures of Childhood Neighborhood Context for Life Course Epidemiologic Studies
Source: PLoS One. 2015 Apr 21;10(4):e0124635. doi: 10.1371/journal.pone.0124635 (PMC4405544; doi:10.1371/journal.pone.0124635)
Supplement: S1 File — (DOCX) [file pone.0124635.s001.docx]

S1 File. Supporting Information Tables for the LIFE Measures of Childhood Neighborhood Context.

Supporting Information Table A. Comparison of LIFE Demographic, Socioeconomic, and Health Variables between Participants with Validated Geocoded Addresses and Participants with Invalid Geocoded or Missing Addresses, for Childhood Address.

| Variable |  | Valid Geocoded Childhood Address N = 693 | |  | Invalid or Missing Childhood Address for Geocode N = 349 | |  |  |
| --- | --- | --- | --- | --- | --- | --- | --- | --- |
|  |  | Mean/N | SD/% |  | Mean/N | SD/% |  | p-value^ |
| Age |  | 27.5 | 6.2 |  | 27 | 6.2 |  | 0.176 |
| Married |  | 207 | 30% |  | 98 | 28% |  | 0.549 |
| Household Size |  | 3.3 | 1.4 |  | 3.2 | 1.5 |  | 0.238 |
| Parity Total Live Births |  | 2 | 1.2 |  | 2 | 1.2 |  | 0.793 |
| Household Income | | 43,197 | 34,993 |  | 42,677 | 37,771 |  | 0.834 |
|  | Household Income % Missing | 51 | 7% |  | 39 | 11% |  | 0.039 |
| Education |  |  |  |  |  |  |  |  |
|  | < High School Diploma | 37 | 5% |  | 20 | 6% |  | 0.991 |
|  | High School Diploma or GED | 76 | 11% |  | 39 | 11% |  |  |
|  | Some College/Tech Training/Associates Degree | 518 | 75% |  | 260 | 75% |  |  |
|  | Bachelor’s Degree or Higher | 62 | 9% |  | 30 | 9% |  |  |
| Employment |  |  |  |  |  |  |  |  |
|  | Missing | 3 | 0.40% |  | 3 | 0.90% |  | 0.811 |
|  | Working | 343 | 49% |  | 180 | 52% |  |  |
|  | Unemployed | 191 | 28% |  | 96 | 28% |  |  |
|  | Out of Workforce | 124 | 18% |  | 56 | 16% |  |  |
|  | Other | 32 | 5% |  | 14 | 4% |  |  |
| Current Subjective Neighborhood Measures | | |  |  |  |  |  |  |
|  | Social Cohesion Scale | 24.3 | 4.9 |  | 23.8 | 5 |  | 0.158 |
|  | Social Disorder Scale | 12 | 4.6 |  | 11.8 | 4.8 |  | 0.547 |
|  | Victimization | 44 | 6.40% |  | 18 | 5.20% |  | 0.443 |
| Length of Residence at Current Address | | |  |  |  |  |  |  |
|  | Mean # of months, including outliers* | 57.9 | 86 |  | 44.8 | 61 |  | 0.005 |
|  | Mean # of months, excluding outliers* | 32.8 | 40 |  | 36.2 | 40.6 |  | 0.214 |
|  | Median # of months | 24 | 53.0† |  | 24 | 53.0† |  | 0.75 |
|  | % Living in current residence 1+ years | 445 | 64% |  | 229 | 66% |  | 0.127 |
|  | % Living in current residence 5+ years | 184 | 27% |  | 87 | 25% |  | 0.098 |
| Current City of Residence | |  |  |  |  |  |  |  |
|  | Detroit | 349 | 50% |  | 151 | 43% |  | 0.045 |
|  | Other | 335 | 48% |  | 189 | 54% |  |  |
|  | Missing | 9 | 1% |  | 9 | 3% |  |  |
| Current (Adult) Health | |  |  |  |  |  |  |  |
|  | CES-D Depressive Symptoms Score | 15.6 | 9.7 |  | 16.1 | 10.4 |  | 0.418 |
|  | Self-Rated Health | 2.8 | 1.1 |  | 2.7 | 1 |  | 0.185 |
|  | Low Birthweight | 94 | 14% |  | 41 | 12% |  | 0.410 |

†Interquartile Range. * Outliers defined as outside the mean +/- 2SD. ^ Statistical tests of difference: Categorical variables assessed with Pearson Chi-Square Test; Continuous variable means assessed with Two Sample T-Test; Continuous variable medians assessed with Wilcoxon Log-Rank Test

Supporting Information Table B. Supplemental Recall Bias Analysis. Interaction Models, for Objective Childhood Neighborhood Measures with either Residential Stability at Adult Address, or Age, Predicting Subjective Childhood Neighborhood Context. LIFE Study.

|  | **Childhood Neighborhood Social Control** | | |  | **Childhood Neighborhood Social Disorder** | | |  | **Childhood Neighborhood Victimization** | | |
| --- | --- | --- | --- | --- | --- | --- | --- | --- | --- | --- | --- |
| **Interaction** | **β** | **SE** | **P** |  | **β** | **SE** | **P** |  | **β** | **SE** | **P** |
| Residential Stability |  |  |  |  |  |  |  |  |  |  |  |
| X Past Neigh Deprivation | -0.004 | 0.003 | 0.309 |  | -0.001 | 0.003 | 0.669 |  | 0.004 | 0.002 | 0.085 |
| X Past Neigh Poverty Rate | -0.043 | 0.034 | 0.204 |  | 0.015 | 0.029 | 0.608 |  | 0.056 | 0.024 | 0.017 |
| X Past Neigh Share Black | -0.004 | 0.017 | 0.817 |  | -0.019 | 0.015 | 0.220 |  | 0.001 | 0.013 | 0.908 |
| Age |  |  |  |  |  |  |  |  |  |  |  |
| X Past Neigh Deprivation | -0.365 | 0.259 | 0.159 |  | 0.029 | 0.218 | 0.895 |  | 0.233 | 0.180 | 0.194 |
| X Past Neigh Poverty Rate | -2.801 | 2.405 | 0.245 |  | -0.026 | 2.039 | 0.990 |  | 2.336 | 1.585 | 0.141 |
| X Past Neigh Share Black | -1.952 | 1.251 | 0.119 |  | 0.474 | 1.107 | 0.669 |  | 0.347 | 0.957 | 0.717 |

Note: Childhood neighborhood operationalized as neighborhood at age 10. Residential Stability is modeled as current length of residence modeled in number of months, excluding outliers (outside mean +/- 2SD). Age is modeled as a dichotomous variable (<30 yr/≥30 yr).N = 624 for most models, and N = 686 for neighborhood deprivation models.

Supporting Information Table C. Comparison of Residential Stability between LIFE Sample, and the US Population of Blacks aged 18-44.

|  | LIFE (geocoded sample) | US Blacks aged 18-44 | US Total Population Aged 18-44 | US Total Population (all ages) |
| --- | --- | --- | --- | --- |
| 5-year residential stability | 27% | 47% | 50% | 65% |
| 1-year residential stability | 64% | 79% | 81% | 87% |

Data for the US Black population from the US Census Bureau, Annual Social and Economic Supplement (ASEC) to the Current Population Survey (CPS), Detailed Tables. Source: http://www.census.gov/hhes/migration/data/cps.html
